# Supplementary material for: Rheological inheritance controls the formation of segmented rifted margins in cratonic lithosphere
Source: Nat Commun. 2021 Aug 2;12:4653. doi: 10.1038/s41467-021-24945-5 (PMC8329282; doi:10.1038/s41467-021-24945-5)
Supplement: Supplementary file 1 — Supplementary Information [file 41467_2021_24945_MOESM1_ESM.pdf]

Supplementary information to

Rheological inheritance controls the formation of segmented rifted margins in cratonic lithosphere

M. Gouiza[1] and J. Naliboff[2,3]

[1] School of Earth and Environment, University of Leeds, Leeds, UK. [2] Department of Earth and Planetary Sciences, University of California, Davis, USA. [3] New Mexico Institute of Mining and Technology, Socorro, New Mexico, USA.

Correspondence and requests for materials should be addressed to M.G (email: m.gouiza@leeds.ac.uk) or to J.N. (email: john.naliboff@nmt.edu).

Note 1: Extension rates, continental breakup, and seafloor spreading in the Labrador Sea

Seismic reflection data along the Labrador margin indicate that the amount of continental extension (L; see Supplementary Table 1) decreases towards the north. This extension was accommodated by major rifting graben and half-graben structures, which were clearly imaged in the southern and central segments of the margin. They are filled by Lower to Upper Cretaceous sediments and provide good constraints on the duration of rifting (ca. 65 Myr: 145 – 80 Ma)[1–3]. On the other hand, the lack of rifting structures and syn-rift sediments in the northern segment hinders any assumption on extension rate.

The present-day crustal architecture, depicted by seismic data and gravity modelling, suggest an asymmetric rifting in the Labrador Sea, which displays wider shelf and necking domains on the Canadian side than on the Greenland side[1–3]. Ref. [4] suggests that the line of breakup was closer to the Greenland side (i.e., upper plate) than the Labrador side (i.e., lower plate), which means that most the syn-rift stretching is preserved on the Canadian side of the basin. Overall, stretching in the Labrador Sea appears to be characterized by slow crustal extension rates then higher mantle exhumation rates.

Various ages of continental breakup were proposed in the Labrador Sea, ranging from Turonian (ca. 92 Ma), based on a stratigraphic unconformity[5], to Palaeocene (ca. 62 Ma), corresponding to the magnetic anomalies of Chron 27[6]. Ref. [1] used seismic reflection lines (to identify the extent of oceanic crust) combined with magnetic chrons[7] to propose a diachronous continental breakup younging northward. They suggest that oceanic accretion started ca. 65.8-64.4 Ma in the south (i.e., Chron 29), ca. 63.3-61.1 Ma in the centre (i.e., between Chron 29 and Chron 27), and ca. 60.5-57.7 Ma in the north (i.e., Chron 26). The latter coincides with the mid to late Palaeocene (ca. 61-56 Ma) flood basalts, which are found around the Davis Strait and attributed to the Iceland plume[8,9].

Seafloor spreading in the Labrador Sea experienced a change in direction from NE-SW to N-S at ca. 60 Ma[10]. Then at ca. 50 Ma spreading rate decreased from 10 to 3-4 mm/yr (half spreading rates) before it completely ceased at the Eocene-Oligocene boundary[10] (ca. 34 Ma).

Note 2: Surface heat flow onshore the Labrador margin

Data from the east Canadian Shield, onshore the Labrador margin. show a noticeable southward increase in surface heat flow[11]. Surface heat flow values range between ca. 22-27 mW/m^2 in the Churchill and Nain provinces in the north, 27-37 mW/m^2 in the Makkovik province in the centre, and 27-47 mW/m^2 in the Grenville province in the south. Variations in heat flow can be explained by changes in crustal radiogenic heat production (i.e., differences in crustal thickness and/or composition), while regional-scale variations are related to changes in lithospheric mantle heat flow[12,13].

The southward increase in surface heat flow onshore Labrador is consistent with the observed increase and decrease in crustal and lithospheric thickness south of the Grenville Front, respectively. Lateral variations in shear velocities across the Grenville Front at depths ranging between 80 and 150 km also suggests changes in temperature (and composition) of the deep mantle lithosphere[14,15]. The Grenville Front was a major suture zone along which the Superior province was subducted toward the SE underneath the Grenville province, giving rise to calc-alkaline arcs[16] (ca. 1.68 to 1.66 Ga). This magmatism has resulted in the depletion of the mantle lithosphere underneath the Grenville domain, such as attested by the distribution of post subduction magmatism which is found on both sides of the Labrador Sea but mostly north of the Grenville Front[17].

Note 3: Initial thermal structure of the numerical experiments

Each of the three segments of the Labrador Sea is represented by a distinct lithospheric structure setup, which is constrained by observations from the Labrador Sea and the Canadian Shield[1] with fixed initial model geometry, temperature structure, mantle rheology, and extension rate (Fig. 2).

The model setup for the north segment has a 200 km thick pre-rift lithosphere with a 20 km thick upper crust and a 15 km thick lower crust. The model setup for the central segment has a 175 km thick pre-rift lithosphere with a 20 km thick upper crust and a 15 km thick lower crust. The model setup for the south segment has a 150 km thick pre-rift lithosphere with a 20 km thick upper crust, a 15 km thick lower crust, and a 15 km thick underplated high density crust.

We assume a weak (fertile) mantle in the north and central segments. which is governed by wet olivine flow low[18], while in the southern segment we use a strong (depleted) mantle lithosphere defined by dry olivine flow law and a weak (fertile) asthenosphere defined by wet olivine flow law[18].

The initial thermal structure of each model is calculated using the following thermal gradient equation[12]:

$$T(z) = T_t + \left(\frac{q_t}{k}\right) z - \frac{(A\ z^2)}{(2\ k)} q_t = q_b + (A\,\Delta z)$$

where T(z) is the temperature at a given depth (z), T_t is temperature at the top of the layer, q_t is the heat flow at the top of the layer, q_b is the heat flow at the base of the layer, A is the radiogenic heat production of the layer, k is the thermal conductivity of the layer, and Δz is the thickness of the layer.

We use surface heat flow of 25, 32.5, and 40 mW m^{-2} for the northern, central, and southern segments respectively, constant radiogenic heat production in the mantle lithosphere (0.01 μW m^{-3}) and lower crust (0.04 μW m^{-3} if felsic and 0.02 μW m^{-3} if mafic), and constant temperature at the lithosphere-asthenosphere boundary (1330 °C). The calculated thermal profiles are shown in Figure 2 and the full thermal parameters are given in Supplementary Table 2.

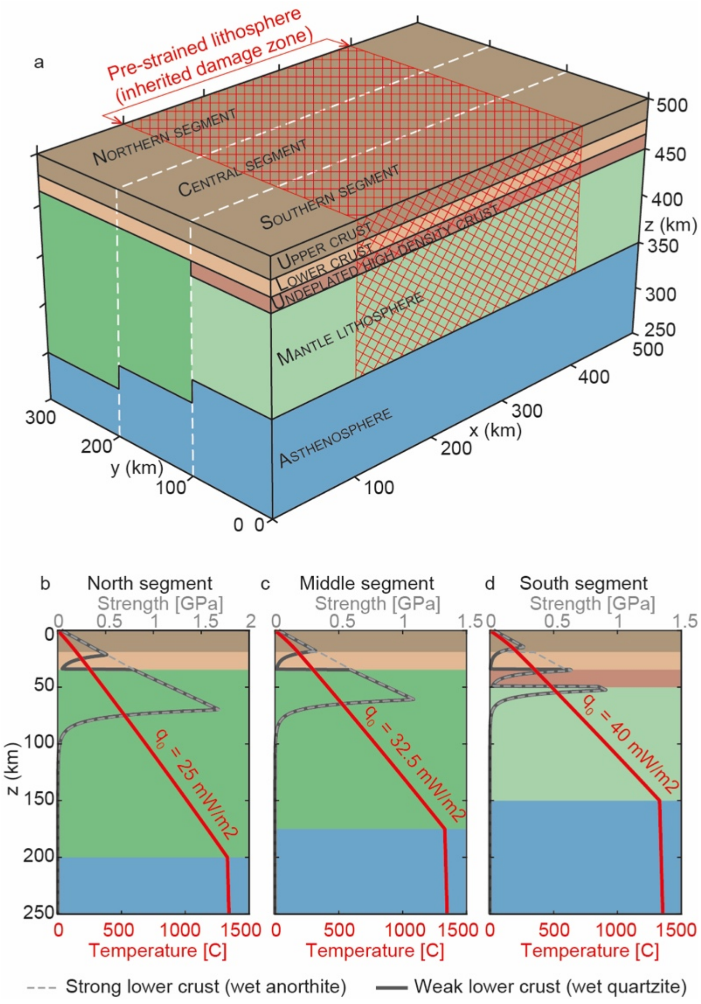

Supplementary Figure 1. Initial setup of the numerical experiments. (a) The numerical experiments assume an initial lithosphere that is 200 km thick in the northern segment, 175 km thick in the central segment, and 150 km thick in the southern segment. The crust in the north and centre is 35 km thick and made of a 20 km thick upper crust and 15 km thick lower crust. In the south, the crust is 50 km thick and made of a 20 km thick upper crust, a 15 km thick lower crust, and a 15 km thick underplated crust. To localise deformation, a pre-strained zone consisting of both plastic and viscous strain is implemented in all models, which is 300 km wide and stretches down to the LAB (lithosphere-asthenosphere boundary). The initial plastic and viscous strains are randomized between values of 0.5-1.5, and both the brittle (cohesion, angle of internal friction) and viscous (pre-yield viscosity) strength are linearly weakened by a factor of 4 over this range. **(b-d)** The initial geothermal gradient (red line) is uniform within each segment but increases southward between the segments and is calculated assuming surface heat flows observed in the Canadian Shield (q_0) and a constant LAB temperature at 1330 °C (see detailed description of initial thermal structure and thermal parameters in Supplementary Note 3 and Supplementary Table 2). The initial strength of the lithosphere depends on the composition of the lower crust, which is governed either by wet quartzite[19] (solid grey line) or wet anorthite[20] (dashed grey line) creep laws. Our models assume a mantle lithosphere governed by dry olivine flow law[18] in the southern segment (light green) and wet olivine flow law[18] in the central and northern segments (dark green). Geochemical evidence suggests that the continental mantle lithosphere beneath cratons may be compositionally depleted in some locations relative to the Asthenospehre[21,22]. While seismic evidence points toward a more complicated two-layer lithospheric mantle structure beneath cratons, here we represent the lithospheric mantle with a single layer that is 50 kg/m3 less buoyant than the asthenosphere at the equivalent

temperature. Future work may explore 3-D simulations with a two-layer lithospheric mantle density and rheology structure, which previous 2-D modelling efforts[23] explored with depleted continental mantle lithosphere densities 50-80 kg/m3 lower than reference mantle densities.

<table>
<tr><th rowspan="2"></th><th rowspan="2">Lines</th><th colspan="7">Continental crust extension</th><th colspan="4">Mantle exhumation</th><th colspan="2" rowspan="2">Total extension rate (mm/yr)</th></tr>
<tr><th>t_{ext} (Myr)</th><th>l_f (km)</th><th>a (km^2)</th><th>l_i (km)</th><th>L (km)</th><th colspan="2">v_{ext} (mm/yr)</th><th>t_{exh} (Myr)</th><th>l_m</th><th colspan="2">v_{exh} (mm/yr)</th></tr>
<tr><td rowspan="2">Northern segment</td><td>1</td><td rowspan="2">?</td><td>92</td><td>1036</td><td>30</td><td>62</td><td>-</td><td rowspan="2">-</td><td colspan="4" rowspan="2">No mantle exhumation</td><td colspan="2" rowspan="2">-</td></tr>
<tr><td>2</td><td>92</td><td>1020</td><td>29</td><td>63</td><td>-</td></tr>
<tr><td rowspan="2">Central segment</td><td>3</td><td rowspan="7">65</td><td>107</td><td>1133</td><td>32</td><td>75</td><td>1.1</td><td rowspan="2">1.6</td><td rowspan="7">15</td><td>57</td><td>3.8</td><td rowspan="2">4.5</td><td>1.6</td><td rowspan="2">2.1</td></tr>
<tr><td>4</td><td>170</td><td>1264</td><td>36</td><td>134</td><td>2.1</td><td>78</td><td>5.2</td><td>2.6</td></tr>
<tr><td rowspan="5">Southern segment</td><td>5</td><td>272</td><td>2935</td><td>59</td><td>213</td><td>3.3</td><td rowspan="5">3</td><td>110</td><td>7.3</td><td rowspan="5">7.0</td><td>4.0</td><td rowspan="5">3.7</td></tr>
<tr><td>6</td><td>298</td><td>4439</td><td>89</td><td>209</td><td>3.2</td><td>108</td><td>7.2</td><td>4.0</td></tr>
<tr><td>7</td><td>349</td><td>4562</td><td>91</td><td>258</td><td>4.0</td><td>90</td><td>6.0</td><td>4.3</td></tr>
<tr><td>8</td><td>168</td><td>1267</td><td>25</td><td>143</td><td>2.2</td><td>85</td><td>5.7</td><td>3</td></tr>
<tr><td>9</td><td>196</td><td>2478</td><td>50</td><td>146</td><td>2.3</td><td>131</td><td>8.7</td><td>3.5</td></tr>
</table>

Supplementary Table 1. Quantification of extension in the Labrador margin, calculated along different margin-perpendicular seismic lines[1] assuming area conservation in 2D. t_{ext}: duration of crustal extension; l_f: final (present-day) length of continental crust along the seismic line; a: continental crust area defined by top basement and Moho; $l_i = a/d_i$ where l_i is the initial (pre-rift) length of continental crust and d_i is the initial (pre-rift) crust thickness (35 km in the northern and central segments, 50 km in the southern segment); $L = l_f - l_i$, is the amount of continental crust extension; $v_{ext} = L/t_{ext}$ is the extension rate; t_{exh}: duration of mantle exhumation; l_m: length of exhumed mantle domain along seismic lines; $v_{exh} = l_m/t_{exh}$ is the exhumation rate.

Parameters			Upper crust	Lower crust	Underplated crust	Mantle lithosphere	Asthenosphere
Density (kg/m^{3})			2700	2850	3000	3250	3300
Radiogenic heat production (µW m^{-3})	*Northern segment*		0.202e-6	0.04e-6 (if wet quartzite flow law) 0.02e-6 (if wet anorthite flow law)	-	0.01e-6	0
	Central segment		0.457e-6		-		
	Southern segment		0.662e-6		0.02e-6		
Thermal conductivity (W K^{-1} m^{-1})			3				
Thermal diffusivity (m^{2} s^{-1})			1.481481e-6	1.40350e-6	1.333333e-6	1.230769e-6	1.212121e-6
Heat Capacity (J kg^{-1} K^{-1})			750				
Thermal expansivity (K^{-1})			2e-5				
Angle of internal friction (°)			30				
Cohesion (MPa)			20e6				
Strain weakening interval			0.5 - 1.5				
Strain weakening factor			4				
Wet quartzite[19]	*Prefactor (Pa^{-n} s^{-1})*		8.57e-28		-		
	Stress exponent		4		-		
	Activation energy (J mol^{-1})		223e3		-		
Wet anorthite[20]	*Prefactor (Pa^{-n} s^{-1})*			7.13e-18		-	
	Stress exponent			3		-	
	Activation energy (J mol^{-1})			345		-	
Dry olivine[18]	*Prefactor (Pa^{-n} s^{-1})*	*Dislocation*	-			6.52e-16	-
		Diffusion	-			2.37e-15	-
	Stress exponent	*Dislocation*	-			3.5	-
		Diffusion	-			1	-

	Activation energy (J mol^{-1}*)*	*Dislocation*	-	530e3	-
		Diffusion	-	375e3	-
	Activation volume (m^3 mol^{-1}*)*	*Dislocation*	-	18e-6	-
		Diffusion	-	10e-6	-
**Wet olivine**[18]	*Prefactor (*Pa^{-n} s^{-1}*)*	*Dislocation*	-	5.33e-19	
		Diffusion	-	1.50e-18	
	Stress exponent	*Dislocation*	-	3.5	
		Diffusion	-	1	
	Activation energy (J mol^{-1}*)*	*Dislocation*	-	480e3	
		Diffusion	-	335e3	
	Activation volume (m^3 mol^{-1}*)*	*Dislocation*	-	11e-6	
		Diffusion	-	4e-6	

Supplementary Table 2. Thermo-mechanical parameters used for the numerical modelling.

**Parameter**			**Value**
$\mathbf{T_{solidus}}$ = $A_1 + A_2*P + A_3*P^2$	*0.0 wt% water in mantle*	A_1 (˚C)	1085.7
		A_2 (˚C MPa^{-1})	1.329e-7
		A_3 (˚C MPa^{-2})	-5.1e-18
	0.05 wt% water in mantle	A_1 (˚C)	940.4
		A_2 (˚C MPa^{-1})	1.332e-7
		A_3 (˚C MPa^{-2})	-5.13e-18
$\mathbf{T_{liquidus}^{lherz}}$ = $B_1 + B_2*P + B_3*P^2$	B_1 (˚C)		1475.0
	B_2 (˚C MPa^{-1})		8.0e-8
	B_3 (˚C MPa^{-2})		-3.2e-18
$\mathbf{T_{liquidus}}$ = $C_1 + C_2*P + C_3*P^2$	C_1 (˚C)		1782.0
	C_2 (˚C MPa^{-1})		4.5e-8
	C_3 (˚C MPa^{-2})		-2e-18
$\mathbf{M_{cpx}}$			0.15
$\mathbf{r_1}$			0.5
$\mathbf{r_2}$ **(Pa^{-1})**			8e-11
$\boldsymbol{\beta}$			1.5

Supplementary Table 3. Parameters used for melt calculations[24]. $T_{solidus}$: solidus temperature; $T_{liquidus}^{lherzolite}$: lherzolite liquidus temperature; $T_{liquidus}$: liquidus temperature; P: pressure; M_{cpx}: mass fraction of clinopyroxene in the peridotite to be molten; r_1: constant in the linear function that approximates the clinopyroxene reaction coefficient; r_2: prefactor of the linear pressure term in the linear function that approximates the clinopyroxene reaction coefficient; β: exponent of the melting temperature in the melt fraction calculation.

Supplementary references

1. Gouiza, M. & Paton, D. A. The Role of Inherited Lithospheric Heterogeneities in Defining the Crustal Architecture of Rifted Margins and the Magmatic Budget During Continental Breakup. *Geochem. Geophys. Geosystems* **20**, 1836–1853 (2019).

2. Keen, C. E., Dickie, K. & Dafoe, L. T. Structural characteristics of the ocean-continent transition along the rifted continental margin, offshore central Labrador. *Mar. Pet. Geol.* **89**, 443–463 (2018).

3. Keen, C. E., Dickie, K. & Dafoe, L. T. Structural Evolution of the Rifted Margin off Northern Labrador: The Role of Hyperextension and Magmatism. *Tectonics* **37**, 1955–1972 (2018).

4. Peace, A. *et al.* An evaluation of Mesozoic rift-related magmatism on the margins of the Labrador Sea: Implications for rifting and passive margin asymmetry. *Geosphere* **12**, 1701–1724 (2016).

5. Balkwill, H. R. & McMillan, N. J. Mesozoic - Cenozoic geology of the Labrador shelf. in *Geology of the Continental Margin of Eastern Canada, Geology of Canada* (eds. Keen, M. J. & Williams, G. L.) vol. 2 31–85 (1990).

6. Chalmers, J. A. & Laursen, K. H. Labrador Sea: the extent of continental and oceanic crust and the timing of the onset of seafloor spreading. *Mar. Pet. Geol.* **12**, 205–217 (1995).

7. Srivastava, S. P. & Roest, W. R. Extent of oceanic crust in the Labrador Sea. *Mar. Pet. Geol.* **16**, 65–84 (1999).

8. Steinberger, B., Bredow, E., Lebedev, S., Schaeffer, A. & Torsvik, T. H. Widespread volcanism in the Greenland–North Atlantic region explained by the Iceland plume. *Nat. Geosci.* **12**, 61 (2019).

9. Storey, M., Duncan, R. A. & Tegner, C. Timing and duration of volcanism in the North Atlantic Igneous Province: Implications for geodynamics and links to the Iceland hotspot. *Chem. Geol.* **241**, 264–281 (2007).

10. Roest, W. R. & Srivastava, S. P. Sea-floor spreading in the Labrador Sea: A new reconstruction. *Geology* **17**, 1000–1003 (1989).

11. Mareschal, J. C. & Jaupart, C. Variations of surface heat flow and lithospheric thermal structure beneath the North American craton. *Earth Planet. Sci. Lett.* **223**, 65–77 (2004).

12. Chapman, D. S. Thermal gradients in the continental crust. *Geol. Soc. Lond. Spec. Publ.* **24**, 63–70 (1986).

13. Mareschal, J.-C. & Jaupart, C. Radiogenic heat production, thermal regime and evolution of continental crust. *Tectonophysics* **609**, 524–534 (2013).

14. Shapiro, N. M., Ritzwoller, M. H., Mareschal, J. C. & Jaupart, C. Lithospheric structure of the Canadian Shield inferred from inversion of surface-wave dispersion with thermodynamic a priori constraints. *Geol. Soc. Lond. Spec. Publ.* **239**, 175–194 (2004).

15. Yuan, H. & Romanowicz, B. Lithospheric layering in the North American craton. *Nature* **466**, 1063–1068 (2010).

16. Gower, C. F. The evolution of the Grenville Province in eastern Labrador, Canada. *Geol. Soc. Lond. Spec. Publ.* **112**, 197–218 (1996).

17. Tappe, S. *et al.* Craton reactivation on the Labrador Sea margins: 40Ar/39Ar age and Sr–Nd–Hf–Pb isotope constraints from alkaline and carbonatite intrusives. *Earth Planet. Sci. Lett.* **256**, 433–454 (2007).

18. Hirth, G. & Kohlstedt, D. Rheology of the upper mantle and the mantle wedge: A view from the experimentalists. in *Geophysical Monograph Series* (ed. Eiler, J.) vol. 138 83–105 (American Geophysical Union, 2003).

19. Rutter, E. H. & Brodie, K. H. Experimental grain size-sensitive flow of hot-pressed Brazilian quartz aggregates. *J. Struct. Geol.* **26**, 2011–2023 (2004).

20. Rybacki, E., Gottschalk, M., Wirth, R. & Dresen, G. Influence of water fugacity and activation volume on the flow properties of fine-grained anorthite aggregates. *J. Geophys. Res. Solid Earth* **111**, B03203 (2006).

21. Griffin, W. L. *et al.* The origin and evolution of Archean lithospheric mantle. *Precambrian Res.* **127**, 19–41 (2003).

22. Yuan, H., Romanowicz, B., Fischer, K. M. & Abt, D. 3-D shear wave radially and azimuthally anisotropic velocity model of the North American upper mantle. *Geophys. J. Int.* **184**, 1237–1260 (2011).

23. Beaumont, C. & Ings, S. J. Effect of depleted continental lithosphere counterflow and inherited crustal weakness on rifting of the continental lithosphere: General results. *J. Geophys. Res. Solid Earth* **117**, B08407 (2012).

24. Katz, R. F., Spiegelman, M. & Langmuir, C. H. A new parameterization of hydrous mantle melting. *Geochem. Geophys. Geosystems* **4**, 1073 (2003).
