# Supplementary material for: Rheological inheritance controls the formation of segmented rifted margins in cratonic lithosphere
Source: Nat Commun. 2021 Aug 2;12:4653. doi: 10.1038/s41467-021-24945-5 (PMC8329282; doi:10.1038/s41467-021-24945-5)
Supplement: Supplementary file 2 — Description of Supplementary Movies [file 41467_2021_24945_MOESM2_ESM.docx]

**Description of Additional Supplementary Files**

File name: Supplementary Movie 1

Description: 2-D numerical model of the northern segment assuming wet quartzite flow law in the upper and lower crust, wet olivine flow law in the mantle, and an extension rate of 5 mm/yr. The upper panel shows the strain rate. The lower left panel shows the distribution of plastic/brittle and viscous/ductile layers. The lower right panel shows the density. Isotherm curves are shown in all three panels.

File name: Supplementary Movie 2

Description: 2-D numerical model of the central segment assuming wet quartzite flow law in the upper and lower crust, wet olivine flow law in the mantle, and an extension rate of 5 mm/yr. The upper panel shows the strain rate. The lower left panel shows the distribution of plastic/brittle and viscous/ductile layers. The lower right panel shows the density. Isotherm curves are shown in all three panels.

File name: Supplementary Movie 3

Description: 2-D numerical model of the southern segment assuming wet quartzite flow law in the upper and lower crust, an anorthite flow law in the underplated crust, dry olivine flow law in the mantle lithosphere, wet olivine flow law in the asthenosphere, and an extension rate of 5 mm/yr. The upper panel shows the strain rate. The lower left panel shows the distribution of plastic/brittle and viscous/ductile layers. The lower right panel shows the density. Isotherm curves are shown in all three panels.

File name: Supplementary Movie 4

Description: 2-D numerical model of the northern segment assuming wet quartzite flow law in the upper crust, wet anorthite flow law in the lower crust, wet olivine flow law in the mantle, and an extension rate of 5 mm/yr. The upper panel shows the strain rate. The lower left panel shows the distribution of plastic/brittle and viscous/ductile layers. The lower right panel shows the density. Isotherm curves are shown in all three panels.

File name: Supplementary Movie 5

Description: 2-D numerical model of the central segment assuming wet quartzite flow law in the upper crust, wet anorthite flow law in the lower crust, wet olivine flow law in the mantle, and an extension rate of 5 mm/yr. The upper panel shows the strain rate. The lower left panel shows the distribution of plastic/brittle and viscous/ductile layers. The lower right panel shows the density. Isotherm curves are shown in all three panels.

File name: Supplementary Movie 6

Description: 2-D numerical model of the southern segment assuming a wet quartzite flow law in the upper crust, anorthite flow law in the lower crust and underplated crust, dry olivine flow law in the mantle lithosphere, wet olivine flow law in the asthenosphere, and an extension rate of 5 mm/yr. The upper panel shows the strain rate. The lower left panel shows the distribution of plastic/brittle and viscous/ductile layers. The lower right panel shows the density. Isotherm curves are shown in all three panels.

File name: Supplementary Movie 7

Description: 2-D numerical model of the northern segment assuming wet quartzite flow law in the upper and lower crust, wet olivine flow law in the mantle, and an extension rate of 10 mm/yr. The upper panel shows the strain rate. The lower left panel shows the distribution of plastic/brittle and viscous/ductile layers. The lower right panel shows the density. Isotherm curves are shown in all three panels.

File name: Supplementary Movie 8

Description: 2-D numerical model of the central segment assuming wet quartzite flow law in the upper and lower crust, wet olivine flow law in the mantle, and an extension rate of 10 mm/yr. The upper panel shows the strain rate. The lower left panel shows the distribution of plastic/brittle and viscous/ductile layers. The lower right panel shows the density. Isotherm curves are shown in all three panels.

File name: Supplementary Movie 9

Description: 2-D numerical model of the southern segment assuming wet quartzite flow law in the upper and lower crust, an anorthite flow law in the underplated crust, dry olivine flow law in the mantle lithosphere, wet olivine flow law in the asthenosphere, and an extension rate of 10 mm/yr. The upper panel shows the strain rate. The lower left panel shows the distribution of plastic/brittle and viscous/ductile layers. The lower right panel shows the density. Isotherm curves are shown in all three panels.

File name: Supplementary Movie 10

Description: 2-D numerical model of the northern segment assuming wet quartzite flow law in the upper crust, wet anorthite flow law in the lower crust, wet olivine flow law in the mantle, and an extension rate of 10 mm/yr. The upper panel shows the strain rate. The lower left panel shows the distribution of plastic/brittle and viscous/ductile layers. The lower right panel shows the density. Isotherm curves are shown in all three panels.

File name: Supplementary Movie 11

Description: 2-D numerical model of the central segment assuming wet quartzite flow law in the upper crust, wet anorthite flow law in the lower crust, wet olivine flow law in the mantle, and an extension rate of 10 mm/yr. The upper panel shows the strain rate. The lower left panel shows the distribution of plastic/brittle and viscous/ductile layers. The lower right panel shows the density. Isotherm curves are shown in all three panels.

File name: Supplementary Movie 12

Description: 2-D numerical model of the southern segment assuming a wet quartzite flow law in the upper crust, wet anorthite flow law in the lower crust and underplated crust, dry olivine flow law in the mantle lithosphere, wet olivine flow law in the asthenosphere, and an extension rate of 10 mm/yr. The upper panel shows the strain rate. The lower left panel shows the distribution of plastic/brittle and viscous/ductile layers. The lower right panel shows the density. Isotherm curves are shown in all three panels.

File name: Supplementary Movie 13

Description: 3-D numerical model with the same constant boundary conditions as the 2-D models of the three segments. Here we use a weak wet quartzite flow law in the lower crust and an extension rate of 5 mm/yr. The upper panel shows the evolution of the 3-D model coloured by density. The lower left and right panels show strain rate coloured slices, with isotherms, through the northern and southern segments, respectively.

File name: Supplementary Movie 14

Description: 3-D numerical model with the same constant boundary conditions as the 2-D models of the three segments. Here we use a strong wet anorthite flow law in the lower crust and an extension rate of 5 mm/yr. The upper panel shows the evolution of the 3-D model coloured by density. The lower left and right panels show strain rate coloured slices, with isotherms, through the northern and southern segments, respectively.

File name: Supplementary Movie 15

Description: 3-D numerical model with the same constant boundary conditions as the 2-D models of the three segments. Here we use a weak wet quartzite flow law in the lower crust and an extension rate of 10 mm/yr. The upper panel shows the evolution of the 3-D model coloured by density. The lower left and right panels show strain rate coloured slices, with isotherms, through the northern and southern segments, respectively.

File name: Supplementary Movie 16

Description: 3-D numerical model with the same constant boundary conditions as the 2-D models of the three segments. Here use a strong wet anorthite flow law in the lower crust and an extension rate of 10 mm/yr. The upper panel shows the evolution of the 3-D model coloured by density. The lower left and right panels show strain rate coloured slices, with isotherms, through the northern and southern segments, respectively.

File name: Supplementary Movie 17

Description: 3-D numerical model with the same constant boundary conditions as the 2-D models of the three segments. The lower crust is governed by the strong wet anorthite flow law in the north and the weak wet quartzite flow law in the centre and the south. The extension rate is 5 mm/yr. The upper panel shows the evolution of the 3-D model coloured by density. The lower left and right panels show strain rate coloured slices, with isotherms, through the northern and southern segments, respectively.

File name: Supplementary Movie 18

Description: 3-D numerical model with the same constant boundary conditions as the 2-D models of the three segments. The lower crust is governed by the strong wet anorthite flow law in the north and the weak wet quartzite flow law in the centre and the south. The extension rate is 10 mm/yr. The upper panel shows the evolution of the 3-D model coloured by density. The lower left and right panels show strain rate coloured slices, with isotherms, through the northern and southern segments, respectively.
